# Intestinal Saturated Long-Chain Fatty Acid, Glucose and Fructose Transporters and Their Inhibition by Natural Plant Extracts in Caco-2 Cells

**DOI:** 10.3390/molecules23102544

**Published:** 2018-10-06

**Authors:** Katharina Schreck, Matthias F. Melzig

**Affiliations:** 1 Freie Universitaet Berlin, Institute of Pharmacy-Pharmaceutical Biology, Koenigin-Luise-Str. 2+4, D-14195 Berlin, Germany; kschreck@zedat.fu-berlin.de; 2 Freie Universitaet Berlin, Institute of Pharmacy-Pharmaceutical Biology, Koenigin-Luise-Str. 2+4, D-14195 Berlin, Germany

**Keywords:** diabetes, Caco-2 cells, plant extracts, uptake inhibition, intestinal transporters, FATP2, FATP4, SGLT1, GLUT2, GLUT5

## Abstract

The intestinal absorption of fatty acids, glucose and fructose is part of the basic requirements for the provision of energy in the body. High access of saturated long-chain fatty acids (LCFA), glucose and fructose can facilitate the development of metabolic diseases, particularly the metabolic syndrome and type-2 diabetes mellitus (T2DM). Research has been done to find substances which decelerate or inhibit intestinal resorption of these specific food components. Promising targets are the inhibition of intestinal long-chain fatty acid (FATP2, FATP4), glucose (SGLT1, GLUT2) and fructose (GLUT2, GLUT5) transporters by plant extracts and by pure substances. The largest part of active components in plant extracts belongs to the group of polyphenols. This review summarizes the knowledge about binding sites of named transporters and lists the plant extracts which were tested in Caco-2 cells regarding uptake inhibition.

## 1. Introduction

As many diseases are triggered by an unhealthy lifestyle, the metabolic syndrome, which is a preliminary stage of type-2 diabetes mellitus (T2DM), is caused by overweight; obesity; tobacco consumption; physical inactiveness; and unhealthy nutrition, especially high levels of saturated long-chain fatty acids (LCFA), glucose and fructose. The metabolic syndrome is accompanied by impaired glucose tolerance, insulin resistance in peripheral tissues, and raised fasting and postprandial glucose and insulin levels. The latter leads to an exhaustion of insulin production in the islets of Langerhans, to dysregulated insulin secretion and to β-cell apoptosis. During the progression of the disorder, the insulin secretion of the pancreas decreases and the metabolic abnormalities increase and turn into a manifest T2DM [1,2].

T2DM causes damage to the heart, blood vessels, nerves, eyes and kidneys in a serious way, which can lead to blindness, limb amputation, heart attack and other endpoints, if not treated appropriately. Not only high glucose and fructose levels, but also elevated saturated fat levels in the blood can impair glucose tolerance and fasting glycemia [2].

**Figure 1:**
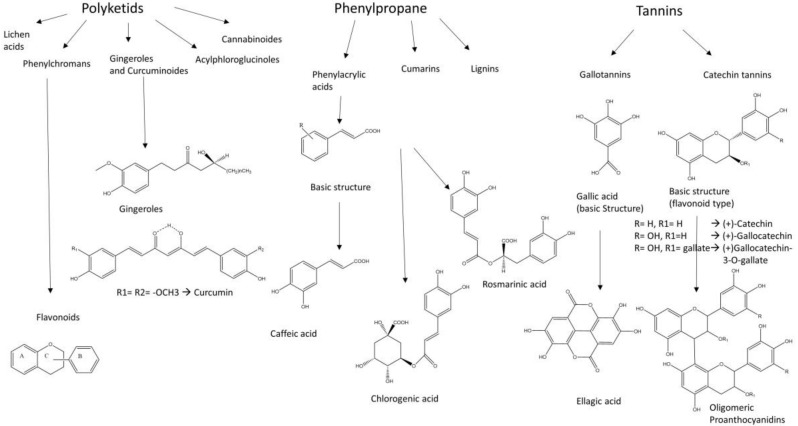
The group of polyphenols consists of different subgroups. This figure only shows polyketids, phenylpropane derivatives and tannins which, in turn, include subgroups. Many of the active substances in plant extracts are derived from the basic structures, which are shown in the illustration.

Civilization-caused diseases spread over the world due to unreasonable lifestyle changes and a lack of information. In 2014, 422 million people were suffering from T2DM and the number is going to raise to twice as much in the next 20 years. T2DM makes up approximately 90% of all diabetes forms worldwide and there is raising incidence for developing the disease in childhood. In 2015, roughly 1.6 million people died of diabetes mellitus [2,3,4,5]. Some traditional medicines contain plant extracts which are used to improve symptoms or stop the progression of the disease. Therefore, research has been done to test plant extracts and pure components for their inhibitory activity on intestinal glucose and fructose transporters using different cell models and detection methods. In contrast, a few natural extracts and some pure substances have been tested to inhibit saturated LCFA transporters in the intestine. With regard to the studies which are listed in this review, the active substances in natural plant extracts mostly belong to polyphenols, such as polyketides (anthocyanins, flavonoids), phenyl propane derivatives (phenyl acrylic acids, lignins) and tannins (catechin tannins, gallotannins), which are structurally shown in Figure 1.

**Figure 2:**
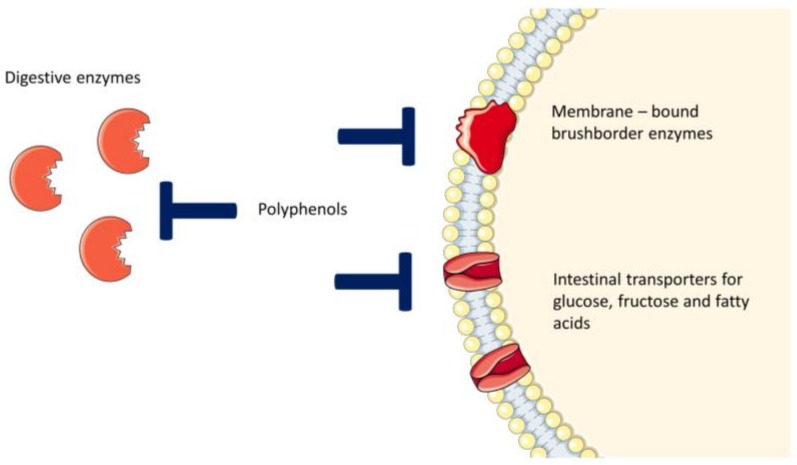
Feasible targets of polyphenols in the intestinal lumen.

The body’s enzymes and intestinal bacteria are able to metabolize some of the active ingredients because they are often conjugated structures linked to sugars, organic acids or other groups. Polyphenols are able to inhibit or to retard glucose uptake by interacting with digestive enzymes, membrane-bound brush border enzymes and apically located transporters [6], as illustrated in Figure 2.

## 2. The Role of Intestinal Fatty Acid, Glucose and Fructose Uptake with Regards to Developing Metabolic Disorders

As known, high loads of specific food components such as monosaccharides and fatty acids, play a crucial role in developing metabolic disorders. Therefore, the suppression or delay of the intestinal saturated LCFA, glucose and fructose absorption into the enterocytes represents a possible target. Decelerating digestion of these food components can improve the health status of the patient and stop the deterioration of the disorder by avoiding high postprandial glucose, fructose and saturated LCFA peaks in the blood [7,8,9,10]. Additionally, the reduction of overweight, which is also related with permanent increased fatty acid blood concentrations, can abolish the progression of the metabolic syndrome and T2DM [9]. Metabolic disorders are very complex and affect the health level of the whole body. Patients with T2DM show an elevated risk to suffer from cardiovascular diseases and early mortality [1,11].

Studies tried to show the relevance of transporters in different models. The intestinal LCFA uptake of fatty acid transport protein 2 (FATP2)-knockout mice has not been observed, but selective FATP2 inhibitors, such as Lipofermata, show 80% attenuation of LCFA uptake across the gut [12,13]. A 48% decrease of FATP4 protein level leads to a 40% reduction of LCFA uptake in isolated primary enterocytes of mice, whereas no change of lipid uptake has been detected in vivo. This observation could be related to the large capacity of the small intestine regarding fat absorption [14]. Röder et al. showed not only a reduced uptake of glucose into enterocytes in sodium-glucose linked transporter 1 (SGLT1)-lacking mice, but also lower serum concentration of glucose and a decreased GIP and GLP-1 secretion after ingestion [15]. Glucose transporter 2 (GLUT2) is present in many organs, e.g. liver, β-cells and kidney, which manage metabolic processes, especially glucose homeostasis. Of course, GLUT2-knockout miceshow sever damage in their metabolic processes and suffer from early mortality. Surprisingly, intestinal GLUT2 absence appeared not to have any influence on intestinal glucose uptake [16]. This observation could be related to a compensating effect of SGLT1 as major intestinal glucose transporter. Experiments with GLUT5 knockout mice showed the relevance of GLUT5 in intestinal fructose uptake. The fructose absorption was reduced by 75% and the serum level decreased by 90% compared to wild mice after fructose ingestion [17]. Table 1 shows the tissue expression of these transporters, which might be relevant when looking for side effects after absorption of inhibitors.

### 2.1. Fatty Acids and Obesity

Fatty acids show multifunctional properties in physiological tissues. They are basic material for membrane biosynthesis, are crucial for transcriptional regulation and exhibit a central source for the provision of energy maintaining the metabolic processes in the human body [7]. Excess of fatty acids leads to accumulation of these food components in non-adipose tissues, such as liver and muscles, which are not designed to store fat in high amounts. Enrichment of fatty acids, especially of saturated LCFAs, in non-adipose tissues entails devastating consequences, in particular, endoplasmic reticulum-stress (ER-stress), and cell dysfunction and apoptosis, designated as lipotoxicity [9,10,12]. With a focus on obesity and nutrition, some food components such as saturated LCFAs and trans-fatty acids are able to trigger inflammatory activity, which plays also an important role in generating insulin resistance, starting mainly in hypothalamus before going down to other tissues and organs [1,8,9,10,11]. The level of inflammation processes due to the activity of cytokines as well as the serum concentration of fatty acids are increased in obese people and can lead to a chronic decrease of insulin secretion by pancreatic β-cells [7,11,21,22].

Especially palmitate and arachidonic acids trigger ER-stress, cell death and inactivation of the insulin receptor-signaling cascade due to increased activation of c-jun-N-terminal kinase (JNK) and inhibition of insulin receptor substrate-1 by a phosphorylation at a critical serine/threonine residue [8,21,23,24,25]. ER-stress is suggested to be linked with the activation of sterol regulatory element-binding protein (SREBP) [7]. Saturated LCFAs initiate a decrease of mitochondrial cardiolipin, which promotes the release of cytochrome C, one of the protagonists in the process of cell apoptosis. Furthermore, metabolites of fatty acids are used in the biosynthesis of ceramides, which are known as lipid signaling molecules and inducers of cell death. Excess of saturated LCFAs, especially palmitate, influences the cell metabolism negatively by altering many cell cascades and processes due to reduced phosphorylation of protein kinase B, elevated diacylglycerol levels and protein kinase C-ɛ activity, decreased activation of phosphatidylinositol-3-kinase and generally an increased cellular oxidative stress in different models [11,21,24]. Additionally, saturated LCFAs are able to influence the expression of glucose transporter 2 (GLUT2) negatively and serve as a substrate of peroxisome proliferator-activated receptor α (PPARα) modulating insulin secretion [21].

In contrast, polyunsaturated fatty acids appear to have a good impact on the body’s fat metabolism [23]. Unsaturated fatty acids including mono- and polyunsaturated fatty acids (MUFA, PUFA), especially omega-3 fatty acids, such as eicosapentaenoic (EPS) and docosoahexaenoic acid (DHA), but also oleate, act as counterparts to their saturated derivatives and are able to prevent induced ER-stress, inflammation and insulin resistance [9,10,22,25].

### 2.2. Monosaccharides and Glycemia

The impact of monosaccharides, especially glucose and fructose, on the development of metabolic disorders and the exact mechanism are still debated and studies show controversial findings [26,27]. Fructose is a component of sweeteners like sucrose and high fructose corn syrup. It is supposed to be one of the main actors of dysregulating fat and carbohydrate metabolism, causing insulin resistance, non-alcoholic fatty liver and hyperuricemia [26]. The liver gathers 70% of oral fructose intake and 15–30% of glucose intake [28,29]. Fructose metabolism promotes and enhances synthesis of lipids in the liver by providing substrates for lipogenesis and activating SREBP-1c [26,27,28,30]. Furthermore, it is suggested that fructose causes hyperinsulinemia, as shown in rats and humans [31,32], and decreased insulin sensitivity in humans [33,34], but the mechanism remains unclear [28]. The negative effects of monosaccharides on metabolism depend on the amount of total energy intake [27,30], which is significant for the accumulation of fat in liver and muscles. Excessive intake of sugars (fructose, glucose, sucrose) accompanied by other energizing sources as existing in patients with increased serum lipid levels, leads more easily to an enrichment of fat in the liver and muscles related to obesity and impaired insulin sensitivity, which generates metabolic disorders [27,30].

Once unhealthy nutrition and lifestyle, including excessive uptake of sugars or saturated LCFAs, has wreaked damage and insulin resistance, the metabolic processes of glucose are restricted, which is leading to additional defects supporting the progression of the metabolic disease. At this point, high blood glucose peaks are even more harmful because they stay for an unphysiologically long duration due to insulin resistance of the main insulin-dependent glucose transporters in peripheral tissues [35]. The irreversible non-enzymatic reaction of proteins with reducing sugars such as glucose, occurs as a product of high unphysiological blood glucose levels for weeks and months. At first, amino groups of endogenous proteins, lipids and nucleic acids react with glucose to a Schiff base and Amadori products which are reversible reaction products. Subsequently, they will be modified to advanced glycosylation end products (AGEs) irreversibly [36], whereby they lose their physiological functions and they are not able to work appropriately, entailing a slow destruction of tissues and organs [36,37]. Many typical symptoms of T2DM and comorbidities take shape, e.g. neuropathy, retinopathy, nephropathy, vascular disease etc. [1,2]. T2DM patients show a two- to three-fold increased risk to die of cerebro-cardiovascular diseases and events, such as heart attack or stroke [2], due to acceleration of atherosclerosis caused by AGE-linked low density lipoprotein (AGE-LDL), which reattaches to tissues inside and outside the vasculature [38,39,40]. The AGEs are also used in diagnosis and therapy control. The glycosylation of hemoglobin type A1c (HbA1c) works as a marker for diabetic complications to check therapy response and compliance [41]. The postprandial increase of the blood glucose concentration depends on the food components and can be expressed as glycemic index. Studies show a preferable effectivity of diets with low glycemic nutrients regarding the control of glycemia, increase of insulin sensitivity and reduction of glycosylated hemoglobin [1].

## 3. Cell Models

Apart from experiments on animals such as rats, mice or rabbits or on animal intestinal tissue compartments, there are existing cell models with human cell lines which are less invasive, well examined and convenient for studying transport mechanisms. The selection for one of the cell lines depends mostly on the duration of proliferation; the type of differentiation regarding transporters, tight junctions and enzymes; specific characteristics of the cell line; availability; or other experimental requirements for the cells. Thirty years ago, Chantret et al. gave a short summary about some of the cell lines and classified them into four groups. The first group included only human colonic adenocarcinoma (Caco-2) cells. They are able to spontaneously differentiate into enterocytes with features of the small intestine within approximately 20 days after confluence and are, to date, one of the most used cell lines for transport studies [42,43,44,45]. Meanwhile, some companies provide over 60 different human adenocarcinoma cell lines derived from tumors at various stages developing different characteristics. Caco-2 cells grow into a polarized monolayer with an apical brush border and brush border-associated hydrolases, such as lactase and alkaline phosphatase [42]. The cell line also expresses transporters, such as fatty acid transport protein 2 (FATP2), FATP4 [46], sodium-glucose linked transporter 1 (SGLT1), GLUT2 and GLUT5 [47]. The expression of SGLT1 varies from the cell bank origin [48]. For exhibiting a homogeneous expression of differentiated traits, clones of Caco-2 cells as e.g. Caco-2/TC7 were cultivated [45]. Cluster determinant 36 (CD36) and fatty acid binding proteins (FABPs) are also present in the intestine [49,50], but their attendance is not investigated for Caco-2 cells yet. However, there is not much knowledge about the role and arrangement of CD36 and FABP in intestinal fatty acids transport. For investigations into cell lines, specific inhibitors, as shown in Table 2, block the activity of transporters whose influence is not intended. It is challenging to screen plant extracts in the presence of all these transport proteins and to assign the inhibitory effect to one transporter. Furthermore, differentiation of Caco-2 cells is time-consuming [51]. For this reason, there are existing methods called cell-high-throughput-screening assay (HTS)-optimizing substance screenings. Black et al. designed a live-cell high-throughput-screening assay with yeast cells of *Saccharomyces cerevisiae* (Desm.) Meyen, 1838 which express *Mus musculus* Linnaeus fatty acid protein 2 (mmFATP2) [52]. Although yeast cells proliferate rapidly, they reconstitute the function of *Homo sapiens* Linnaeus, 1758 fatty acid transport proteins (hsFATPs) [51]. Zhou et al. created a HTS with recombinant hsFATP1, hsFATP4 and hsFATP5 expressed in human kidney embryonic 293 (HKE293) cells [51]. A yeast-based screening system with cells of *Saccharomyces cerevisiae* (Desm.) Meyen, 1838 expressing hsGLUT5 has been generated to facilitate screening assays for inhibitors [53]. The question remains whether cells of *Saccharomyces cerevisiae* (Desm.) Meyen, 1838 and HKE cells can represent the basic character of human enterocytes.

## 4. Transport Mechanisms and Binding Sites of Intestinal Saturated LCFA, Glucose and Fructose Transporters

The subsequent analysis of the binding sites of LCFA, glucose and fructose transporters plays a crucial role in understanding the interactions between transporter and inhibitor and in identifying substances with inhibitory activity.

### 4.1. Intestinal Long-Chain Fatty Acid Transporters

LCFA (>12 C-atoms) [54] transport can be differentiated into non-protein-mediated and protein-mediated transit. Passive diffusion of LCFAs mainly occurs at high intestinal concentrations of the un-ionized substrate, whereas ionized LCFAs and low physiological concentrations of un-ionized LCFAs require protein-mediated permeation [62]. Three classes of fatty acid transport proteins have been identified: plasma membrane FABP (FABP*_pm_*), fatty acid translocase (FAT = scavenger receptor CD36) and the family of FATP1-6 [62,63,64].

**Figure 3:**
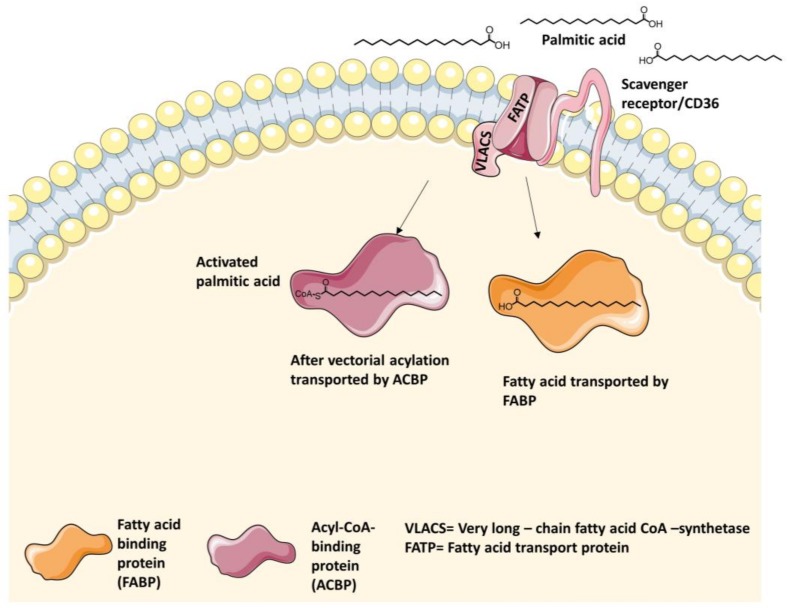
Simplified illustration of the proteins which are involved in fatty acid transport into the enterocyte. Yet it is not clarified whether a first docking of the LCFA onto cluster of differentiation 36 (CD36) is required to initiate the transport process. The proteins are working in a complex whereby FATP seems to be the main actor. The transportation of the LCFA into the cell by FATP is coupled to the vectorial acylation to start cleavage by very long-chain acyl-CoA-synthetase (VLACS). Acyl-CoA-binding protein (ACBP) and FABP transport the LCFA-Acyl-CoA and the incorporated LCFA within the cell stimulating a concentration gradient, which enhances the transport.

Stahl et al. presumed that cluster of differentiation 36 (CD36), FATP, (V)LACS ((very) long-chain acyl-CoA-synthethase) and FABP, which are shown in Figure 3, are arranged in a complex to result in more effective transportation of LCFAs through the cell membrane [65,66].

Different types of fatty acid transport proteins have different functions and not all of them are suitable to work as a target for inhibitors of intestinal fat absorption. Primarily, FABP appears to modulate intracellular lipid homeostasis and therefore substrates are not able to reach it easily from the extracellular compartment [50,67]. CD36 is a ubiquitous protein with various functions, e.g. facilitating LCFA uptake in the heart and adipose tissues in rodents. Noushmehr et al. discussed the role of CD36 as a modulator of insulin secretion and fatty acids uptake into β-cells [68]. Sulfo-N-succinimidyl oleate is an irreversible inhibitor of the transport protein [68,69]. Recent studies showed the essential influence of intestinal CD36 in regulating lipid-mediated satiety [49] and the release of gut peptides [69]. It probably supports intestinal LCFAs uptake.

Many surveys implicate the FATP family as responsible for the main LCFAs uptake into cells [64]. Within the FATP family, six fatty acid transporters have been identified and they belong to the solute carrier family 27 (SLC27). FATPs are labeled with the prefix that refers to their origin (hs = *Homo sapiens* Linnaeus, 1758, mm = *Mus musculus* Linnaeus, 1758 etc.) and a number as the suffix (1–6) [64]. The LCFA transporter with the highest incidence in the intestinal system is still debated. The most represented opinion supported FATP4 [62,64,65,70], but since there was no effect of potential inhibitors in vivo, Black et al. proposed FATP2 as a major intestinal LCFA transporter recently [13]. However, the ineffective in vivo studies of FATP4 inhibitors could relate to the inactivation of the inhibitors by the gut microbiome and/or to the large capacity of the small intestine regarding fat absorption [71].

The crystal structures of the hsFATP1-6 are unknown, but some elements were identified, especially for hsFATP1. In mammals, the FATP structure is highly conserved. The identity rate of 92.2% between mmFATP4 and hsFATP4 is higher than between transporters sharing the same mammalian origin. In humans, the highest orthologous identity of 60.3% exists between hsFATP1 and hsFATP4 [64,72]. Wu et al. showed a hormone-induced translocation of FATP1 by insulin to the plasma membrane in adipocytes and skeletal muscle cells, whereas the translocation could not be shown for FATP4 [72].

In contrast to hydrophilic transporters which usually cross the membrane several times to build a three-dimensional passage for the substrates, the FATP forms at least one transmembrane segment, but multiple membrane-associated domains. Additionally, Richards et al. showed in mice that FATP1 exists as a monomer and homodimer [62,73]. The dimerization of FATP1 probably plays a crucial role in LCFA transport, but it is unclear which conditions have to be complied to form the dimer [74]. FATP1 crosses the membrane near the N-terminus, which is facing the extracellular side and directing three integral domains up to amino acid 190. The segment between 191–257 is not membrane-associated, faces the cytosolic side of the cell and contains 11 amino acids (IYTSGTTGXPK). This motif is consistent in proteins, which either have an interaction side with ATP or catalyze reactions such as acyl-CoA-synthetases, proceeding through adenylated intermediates. Residues 258–475 are located on the peripheral side of the membrane facing the cytosol. The last amino acid sequence of the C-terminus, which contains amino acids 476–646, is not membrane-associated and is turned toward the cytosolic side of the cell as well [62,73]. The structure topology of the outer binding pocket resembles a lipocalin motif, which appears in many transporters with lipophilic substrates [63,75,76]. Many hydrophobic domains are expressed in the segment of the N-terminus that is currently the only part of the transporter with a predicted α-helical structure and is responsible for the substrate docking. The binding site for either ATP or adenylated intermediates supports the acceptance of the subsequent esterification. This vectorial acylation belongs to the transport process of LCFAs and very long-chain fatty acids (VLCFAs), either as an additional associated unit or as a bifunctional transport protein [62,65,73,77], which could also be shown for *Saccharomyces cerevisiae* (Desm.) Meyen, 1838 [78]. VLACS forms fatty acid-CoA derivatives and therefore is able to improve the fatty acid transport due to stimulation of a concentration gradient [54], as shown in Figure 3. The hypothesis of vectorial acylation as a requirement for the metabolism of LCFAs was first put forward by Overath et al. 1969, when they started experiments with *Escherichia coli* (Migula, 1895), Castellani and Chalmers, 1919 [13]. The exact mechanism of protein-mediated LCFA trafficking into the cell is still not clarified by now.

#### 4.1.1. Fatty Acid Transport Protein 2 (FATP2) Inhibitors

Trials with yeast cells which expressed human FATP2 showed different motifs generating interactions between inhibitors and FATP2. The inhibitor is suggested to exhibit a notable lipophilic part as a long aliphatic hydrocarbon chain (cetrimonium bromide and benzalkonium chloride) or a tricyclic core. The tricyclic motif can vary and show a phenothiazine element (perphenazine, perciazine, chlorpromazine, thioridazine, and flufenazine), a cyclobenzaprine with a seven-membered ring instead of the six-membered ring in the center of the core (clomipramine, methiothepin maleate, and cyclobenzaprine) or simply an anthralin structure [46]. No study was performed to show the impact of FATP2 inhibitors on FATP4 performance. FATP2 is also present in the liver and kidney. Consequently, after absorption, the FATP2 inhibitor can cause hypertriglyceridemia, which has been observed as a side effect of antipsychotic drugs such as phenothiazine [46]. Black et al. identified grassofermata as specific and lipofermata as a non-competitive–specific FATP2 inhibitor whose structures are shown in Table 2 [13,79]. Both show inhibitory effects on LCFA and VLCFA uptake into pancreatic β-cells, hepatocytes, myocytes and enterocyte. Accordingly, they are preventive against the accumulation of fatty acids and palmitate acid-induced cell apoptosis [13].

#### 4.1.2. Fatty Acid Transport Protein 4 (FATP4) Inhibitors

FATP4 is supposed to be mainly located in the skin and in the gut. The inhibitory effect of 4-aryl-3,4-dihydro-pyrimidin-2(1H)-ones, whose basic structure is shown in Table 2, is selective for FATP4 over FATP2 and FATP5. Blackburn et al. showed the impacts of different molecule modifications by synthesizing derivatives with various residues. The most effective structure appeared to exhibit a lipophilic ester at position five and a substitution at the para-position, most suitable for NO_2_ or CF_3_. The residues building the ester are preferably unsaturated and long aliphatic chains [54]. Unfortunately, the inhibitory effect was observed in vitro, but did not appear in vivo [13,54].

### 4.2. Intestinal Glucose Transporters

The responsible transporters for the intestinal glucose uptake are SGLT1 and GLUT2 [6], whose characteristics are listed in Table 3 [18,56,80,81,82].

**Figure 4:**
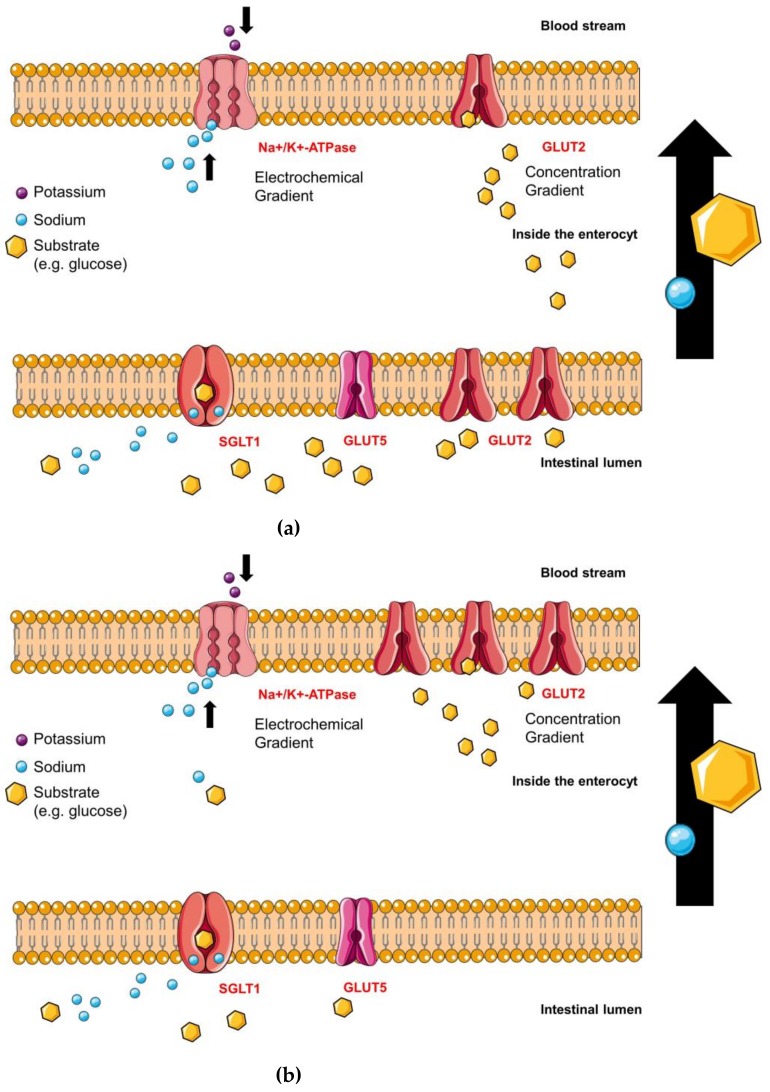
(**a**) At low luminal glucose concentrations, GLUT2 is located on the basolateral side of the cells and it facilitates glucose release into the blood. The sodium-potassium adenosine triphosphatase (Na^+^/K^+^ ATPase) maintains the electrochemical gradient, which is required for the appropriate performance of SGLT1; whereas GLUT2 and GLUT5 work due to the glucose and fructose concentration gradient respectively. (**b**) At high luminal glucose concentrations, GLUT2 is trafficking from the basolateral to the apical side supporting the glucose uptake into the enterocyte.

SGLT1 is located apically, whereas GLUT2 is situated basolaterally and facilitates glucose and fructose transport into the blood stream [82]. Experimental results have indicated that SGLT1 is not the only pathway for glucose into the enterocyte. When the luminal glucose concentration exceeds a specific level in the gut, GLUT2 is able to move to the apical side of the enterocyte supporting the uptake of monosaccharides into the cell shown in Figure 4a and Figure 4b [56]. The GLUT2 translocation is supposed to be dependent on depolarization and Ca^2+^-entry due to activation of SGLT1 [6,80,83,84,85,86]. Affleck et al. were able to demonstrate the evidence of GLUT2 proteins at the apical membrane of the enterocytes immunocytochemically [87]. On the other side, studies show normal results after an oral glucose tolerance test in knockout mice (without GLUT2) and Fanconi Bickel syndrome (FBS) patients (mutation in GLUT2) and they do not exhibit any differences in glucose absorption to wild type mice or healthy humans [82], concluding that GLUT2 inhibitors might not be as effective as SGLT1 inhibitors in vivo.

#### 4.2.1. Sodium-Glucose Linked Transporter 1 (SGLT1)

**Figure 5:**
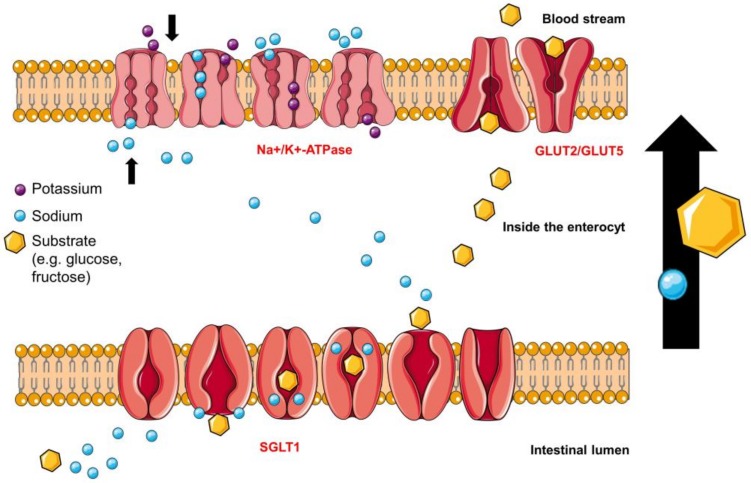
Visualization of the different conformational states of the transporters SGLT1, GLUT2/GLUT5 and of the Na^+^/K^+^-ATPase, which maintains the sodium-electrochemical gradient. At first, sodium docks onto extracellular binding sites of the transporter to cause a conformational change, which facilitate fitting of either glucose or galactose as a substrate into the transmembrane binding site. The outward-occluded binding site turns into an inward-occluded stage. The transporter opens to the inward site and releases the sodium and the substrate into the cell. The process finishes when the inward-facing ligand-free conformation turns into the outward-facing ligand-free position. GLUT2 switches between two conformational states, the inward- and outward-facing conformation, which is called ‘rocker switch’ and is consistent in the major facilitator superfamily.

The amount of SGLT1 proteins is fourfold upregulated in the gut of patients with T2DM and the absorption of monosaccharides from the gut is threefold faster compared to healthy people [6]. SGLT1 is an active, sodium-dependent cotransporter of glucose in a stoichiometry of 2:1. Two ions of sodium, pursued by one molecule of glucose, follow an electrochemical sodium gradient maintained by the Na^+^/K^+^-ATPase, which is located on the basolateral side of the enterocytes, as illustrated in Figure 4a,b [81]. SGLT1 belongs to the SLC5-family and is expressed in several tissues and organs, e.g. small intestine, heart, kidney etc. [18]. The proper mechanism of sugar uptake into the cell undergoes at least six different kinetic states as shown in Figure 5 [88,89]. The principle of Na^+^/K^+^-ATPase and GLUT2 function is also shown in Figure 5, whereby GLUT2 shows a simplified performance [53] compared to SGLT1. The turnover rate (TOR) characterizes the cycle of conformational changes and is defined as the average number of complete cycles performed by a single cotransporter per second [90].

The precise crystal structure of hsSGLT1 has not been totally solved yet, but for vSGLT, which is the bacterial homolog from *Vibrio parahaemolyticus* (Fujino et al., 1951) Sakazaki et al., 1963 [91,92]. Although the known crystal structures of vSGLT, bacterial leucine transporter (LeuT), sodium-benzylhydantoin transport protein from *Microbacterium liquefaciens* (Collins et al., 1983) Takeuchi and Hatano 1998 (Mhp1) and glycine-betaine transporter from *Corynebacterium glutamicum (*Kinoshita et al., 1958) Abe et al., 1967 (BetP) belong to a different gene family, they share the same core domain which includes transmembrane segments (TM) 1–10, and the same structural fold [93]. The crystal structures of vSGLT and LeuT are used as tentative models in combination with alignments for structural and modeling investigations of hsSGLT1. Both transporters, hsSGLT1 and vSGLT, share 32% identity and 75% similarity in their amino acid sequences [82,88]. HsSGLT consists of a sequence of 664 amino acid residues which are arranged in 14 transmembrane α-helices, termed TM-1–13, and contain 13 loops [18,89]. The N-terminus as well as the C-terminus face the outside of the cell [82]. The renumbering of the common model TM-1–13 is based on the crystal structure of the LeuT family to simplify comparisons between structural family members [82]. The stoichiometry of sodium/substrate transport is 2:1 for hsSGLT1, LeuT and BetP and 1:1 for vSGLT and Mhp [93]. Intra- and intermolecular disulfide bonds stabilize the three-dimensional structure of proteins and support the formation of binding vestibules as existing in hsSGLT1 [94]. The known intramolecular disulfide bond in hsSGLT1 connects C255 and C511 [95] and, as proposed by Sasseville et al., hsSGLT1 generates an intermolecular disulfide-bridged homodimer via C355 in its natural tissue [94,96]. In rabbit SGLT, three intramolecular bonds were investigated, proposing the existence of more linked loops in human SGLT [94]. The segment between TM12 and TM13 starts at the intracellular part of the membrane, but a component of the amino sequence was determined to be located extracellularly. Therefore, experiments of Sasseville et al. confirmed a reentrant in loop 13 [96]. To date, it is not clarified what the reentrant loop between TM12 and TM13 exactly looks like. The TM12 segment reaches the cytosolic room and either it crosses the membrane once and enters TM13 from the extracellular side or it crosses the membrane twice to enter TM13 from the intracellular side [94,96]. M. Raja et al. used the classical competitive SGLT1 inhibitor phlorizin, whose structure is shown in Table 2, as a model to determine the binding structures of SGLT1. The high extracellular affinity and low intracellular inhibition activity of phlorizin, especially of the aglycone which is supposed to interact with the loop between TM12 and TM13, also support the hypothesis of an extracellularly located loop between TM12 and TM13 as part of the binding pocket for phlorizin as well as for glucose [94,97]. Conversely, many studies have identified the location of the sodium binding sides in the half of the N-terminus [94]. Another newly identified substance LX2761, whose structure is shown in Table 2, appears to act as a selective SGLT1 inhibitor in vivo [57].

##### Sodium-Binding Site

Two sodium-binding sites, Na1 and Na2, are proposed for hsSGLT1. There has been evidence that the first sodium ion binds to Na2 and the second sodium ion to Na1 [89]. The binding sites are characterized by chemical groups of the amino acids and by their distances to the sodium-ion, which are shown in brackets. The subsequent binding distances might be relevant for inhibitors, which target the sodium-binding sites. Na2 site of hsSGLT1 corresponds with the Na2 site of vSGLT. The carbonyl oxygens of A76 (<4 Å), I79 (<4 Å), S389 (<4 Å) and S393, the side chain hydroxyl from S392 (5 Å) and position D204 of the transporter proteins are coordinating with the sodium molecule [88,89]. The Na1 site of LeuT corresponds to the Na1 site of hsSGLT1 and overlaps with the suggested sugar-binding pocket. In hsSGLT1, the carbonyl oxygen of H83, the aromatic groups of Y290 and W291 are suggested to coordinate with both sodium and sugar because the bonds are flexible and dynamic, not static. E102 belongs to the sugar-binding site and is involved in the sodium binding with its carboxylate group as well as the carbonyl oxygen of N78. Binding interactions are also identified between sodium and H83 (2.5 Å), N78 (2.5 Å), E102 (7.5 Å), Y290 (3.3 Å) and W291 (6.3 Å) [89].

##### Monosaccharide-Binding Site

To date, known coordinating sites for D-glucose are H83, N78, E102, K321, W289, Y290 W291 and Q457 [89]. As described previously, the glucose-binding pocket is partially overlapping with the Na1 interaction site. Glucose-binding sites are also proposed to be located around helices 10–13. Experiments with chimeras of SGLT1 and truncated proteins have shown that residues 381–662, especially amino acids at positions 457, 468 and 499, are crucial for monosaccharide binding. The loop between 12 and 13 contains not only a binding site for phlorizin, but also for glucose. Studies with mutants suggest a correlation between amino acids C345, C351, C355 and C361 and the affinity of D-glucose binding [94]. W289, Y290 and W291 build a formation of aromatic residues, which are essential by influencing the affinity of glucose binding. Residues H83 and N78 coordinate with O2, E102 with O3, W291 with O4, and Q457 with O5/O6 and form a binding pocket for the substrate. The H-bond between N78, which is also interacting with K321 and Y290, enhances the electrostatic potential of the aromatic system and influences the interaction of Y290 and glucose positively [88,89].

##### Phlorizin-Binding Site

Phlorizin, whose structure is shown in Table 2, has been known for its competitive inhibitory activity on SGLT1 and it has been used in cellular transport studies for decades [55] The substance interacts with both the phenol- and the glucoside-(sugar) binding site of SGLT1 [98]. Phlorizin is not used as a drug treatment for inhibition of SGLT1 because the substance is hydrolyzed easily to phloretin by gut enzymes such as lactase-phlorizin-hydrolase (LPH), which are located in the brush border membrane (BBM). Consequently, phlorizin shows a short acting time [99]. Additionally, it has a stronger inhibiting activity on SGLT2 than on SGLT1 and therefore it is the antecedent of the modern SGLT2 inhibitors, which act mainly in the renal system [97]. The loop between TM12 and TM13, which is located extracellularly, has relevance for binding the aglycone of the inhibitor phlorizin, but also contains a binding site for sugar [94,96].

If the aglycone of an inhibitor is coupled to another sugar residue than glucose, the affinity to the binding site will be reduced [100]. Furthermore, the sugar residue is supposed to be in its β-d-pyranose form and the OH-groups in position one, two, three or six are required for proper binding. Otherwise the binding affinity decreases by a factor of five to >200 [82]. Most of the bonds between the transporter and the aglycone of phlorizin are hydrogen bonds. The 4-OH group of ring B interacts with R602, whereas the 4-OH group of ring A is coordinated with D611. The 6-OH-group of ring A is organized with L606–F609 [97] and residues L606–D611 (LFCGLD) are designated as phlorizin-binding domain (PBD). The β-d-glucoside of phlorizin interacts with the monosaccharide-binding sites of SGLT1. The 2-OH and/or 3-OH of the glucose moiety coordinates with K321 and E102, 4-OH with F101 and 6-OH with Q457 [97].

#### 4.2.2. Glucose Transporter 2 (GLUT2) Glucose-Binding-Sites

At low intestinal glucose and fructose concentrations, glucose and fructose uptake into the enterocyte by SGLT1 and GLUT5 respectively on the apical side entails downhill glucose and fructose transport into the blood by GLUT2 on the basolateral side [101]. As mentioned previously, GLUT2 is able to move to the apical side to support glucose and fructose uptake at high luminal concentrations, as shown in Figure 4a and Figure 4b [101,102,103]. The crystal structure of hsGLUT2 is not identified yet, but it is proposed to consist of 524 amino acids and to possess 12 transmembrane segments, forming an aqueous pore [104] with two subunits [105]. The larger subunit is assumed to shape the active binding site. Coordinating amino acids with glucose are S145, K146, P149, S150, L153, S233, L234, Y266, D267, T270, N274, R277, L452, C453, and Q455 [105]. Phloretin, whose structure is shown in Table 2, is widely used in research studies as a relatively specific GLUT2 inhibitor [56,59,60]. Additionally, the substance is able to impede translocation of GLUT2 [106]. Other potent but not specific inhibitors of GLUT2 are glipizide, dihydrochalcone, glaucine and phosphatidylcholine [105].

### 4.3. Intestinal Fructose Transporters

Fructose uptake into cells is insulin-independent [61] and is managed by transport proteins which facilitate passive diffusion [53]. GLUT2 transports glucose and fructose as listed in Table 3 and was already discussed under 4.2.2. Primarily, GLUT5 transports fructose into the enterocyte at the apical side, whereas GLUT2 facilitates fructose transport into the blood stream at the basolateral side of the cell [107,108]. Although GLUT5 belongs to the GLUT family, it appears to be specific for fructose transport [107].

#### 4.3.1. Glucose Transporter 2 (GLUT2) Fructose-Binding Sites

Instead of the amino acid sequence of glutamine-leucine-serine (QLS-motif) in high affinity glucose transporters, such as GLUT1, GLUT3 and GLUT4, the amino acid sequence of histidine-valine-alanine (HVA-motif) is conserved in fructose transporters, such as GLUT2 and GLUT5 [109,110,111]. GLUT2 mainly transports fructose in its furanose form [112]. Research has been done to clear the binding sites of the monosaccharides [113], however it is still not known which exact amino acids of the transporter are involved [112]. Studies trying to identify binding sites to characterize facilitated fructose transport in the human body, have proposed that amino acid I306 plays a crucial role in fructose selectivity for GLUT2 and I321 for GLUT5 [104].

#### 4.3.2. Glucose Transporter 5 (GLUT5)

The crystal structure of hsGLUT5 is not identified yet, but it is supposed to contain 501 amino acids and 12 transmembrane segments [108]. GLUT5 also performs the ‘rocker switch’ [53] as mentioned for GLUT2 in Figure 5. Via the identified crystal structures of the mammalian GLUT5 from *Rattus norvegicus* Berkenhout, 1769 (rGLUT) and *Bos taurus* Linnaeus, 1758 (bGLUT) [114] and due to their ~81% sequence identity to hsGLUT5 [114], many investigations started to model binding sites of hsGLUT5. Tripp et al. suggested an interaction between S72, S76 in TM2 and F424, L428 and F432 in TM11 forming the substrate-binding cavity. The change of amino acids, particularly in bulkier residues, can entail repositioning of TM2 and TM11 into the inward facing conformation [53]. Nomura et al. proposed the essential involvement of TM7 and TM10 in the formation of a substrate gate, especially between residues Y382 in TM10 and I295 and V292 in TM7 in the outward-facing conformation [114]. The binding sites of hsGLUT5 and hsGLUT1 are closely related. Furthermore, human GLUT1 and bovine GLUT5 share only 43% identity, but their crystal structures of the inward-facing conformation superimpose very well as shown by Nomura et al [114]. Similar binding residues between hsGLUT1, bGLUT5, rGLUT5 and hsGLUT5 are suggested. In hsGLUT5, conserved amino acids include I170 (169 in bovine GLUT5), I174 (173), Q167 (166), Q288 (287), Q289 (288), N325 (324) and W420 (419) [108,114]. Especially, tryptophan at position 420 (419 in bGLUT5) plays a crucial role in substrate specificity [114]. George-Thompson et al. showed ligand specificity caused by A396 in hsGLUT5 [61]. Other residues lining the substrate cavity in hsGLUT5, but not conserved in hsGLUT1 and possibly responsible for fructose specificity, are Y32 (31 in bGLUT5), H387 (386), H419 (418), A396 (395) and S392 (391) [108]. Although GLUT7 transports both D-glucose and D-fructose, it is the closest isoform to GLUT5 [114] and therefore Ebert et al. formed GLUT5–GLUT7 chimera to discover the indispensable amino acids for specific fructose uptake of GLUT5. The research group showed a decrease of fructose uptake between 30–80% due to the change of amino acids S41T, L168V, I170V, I174V, V293I, A323V, C331T, A362V, A364L, T368R, A388S, L398V and a decrease below 30% as a result of the switch of amino acids V36L, Q167E, T171I, Y297N, V326I, A332S, V333A, V384I, I399R, P409R, G415D, L428F [108]. A consistent characteristic feature of many transporters in the major facilitator family are salt-bridges between different transmembrane domains. In the mammalian GLUT5, there are only existing salt-bridges far from the cavity site in the outward-facing conformation, including interactions between E151 in TM4, R97 in TM3 and R407 in TM11 and between E400 in TM10, R158 in TM5 and R340 in TM9 [114]. Rubusoside and epicatechingallate show inhibitory activity for GLUT5 as well as for GLUT1 and other transporters [53]. Potent and specific GLUT5 inhibitors are N-[4-(methylsulfonyl)-2-nitrophenyl]-1,3-benzodioxol-5-amine (MSNBA) [53] and astragalin-6-glucoside [61]. Their structures are shown in Table 2.

##### Rubusoside-Binding Site

Whereas rubusoside (CAS: 64849-39-4), which is a transfructosylated steviol (diterpen) glycoside and gained from *Rubus suavissimus*, S. K. Lee inhibited both GLUT1 and GLUT5, astragalin-6-glucoside from *Phytolacca americana* L. acts as specific GLUT5 inhibitor and is structurally shown in Table 2 [61]. George-Thompson et al. identified some of the responsible binding sites of rubusoside in GLUT5 using a homology model of hsGLUT5, which is based on the crystal structure of GLUT1. Interactions due to polar contacts include Q288, Q289 in TM7, N325 in TM8 and S392 in TM10. Although many residues are conserved in GLUT1 and GLUT5, rubusoside docks differently into the cavity of both transporters. In GLUT5, it appears to dock in a curved conformation ~13 Å long [61].

## 5. Plant Extracts with Inhibitory Activity on Intestinal LCFA, Glucose and Fructose Transporters

Traditionally used plants whose extracts have shown inhibitory activity on intestinal glucose and fructose transporters, are listed in Table 4 and Table 5. The discussed active substances are polyphenols and polysaccharides. Research into the inhibitory activity on intestinal saturated LCFA transporters has been done more for pure substances than for plant extracts. Table 6 shows β-glucan plant extracts with inhibitory effects on intestinal LCFA transporters. Nearly all investigations of fatty acid transport inhibition by polyphenolic plant extracts are refering to the reduction of cell uptake into adipose or muscle tissues. For example, Feng et al. studied the corrective effect of the pollen of *Typhae angustifolia* L. extract to the insulin cascade, improving insulin sensitivity in mouse myoblasts [115].

## 6. Polyphenols

Polyphenols belong to the secondary plant compounds, because they are not involved in the primary metabolism of the botanicals. These substances are presumed to have various positive effects such as antihyperglycemic, antibacterial, antiviral and antiproliferative properties on human health and therefore they have constituted the focus of many investigations of new or supporting therapy options for diseases such as cancer and T2DM [149,150,151,152].

Referring to the analytical results of the studies listed in Table 4, Table 5 and Table 6, discussed polyphenols in plant extracts, which show inhibiting activity on intestinal LCFA and monosaccharide transporters, are flavonoids, phenyl acrylic acids and tannins as shown in Figure 1. The flavonoids, which are substantial for the hypoglycemic effects are deduced from the flavan substructure, as are the chalcone derivates phloretin and phlorizin whose structures are shown in Table 2 [153,154]. The flavonoles quercetin, fisetin and myricetin act as potent non-competitive inhibitors of GLUT2 expressed in *Xenopus laevis* Daudin, 1802 oocytes and decrease glucose and fructose uptake into the cells [155]. Tannins, especially (−)-epigallocatechin gallate, (−)-epigallocatechin and (−)-epicatechin gallate showed inhibitory activity for both transporters SGLT1 and GLUT2 [156].

The intestinal transporters for LCFAs, glucose and fructose are not only located in the intestinal cells, but also in many other human tissues (e.g. GLUT2 in pancreatic β-cells) as listed in Table 1. Therefore, the interacting mechanism could be transferable in some cases, which can help to predict the side effects of absorbable polyphenols. After absorption, gallated catechins from green tea such as epigallocatechin-3-gallate are able to block the glucose uptake into peripheral cells in vivo and can evoke secondary hyperinsulinemia [157,158].

## 7. Intestinal Metabolism of Polyphenols

The metabolism and modification of polyphenols, especially of flavonoids, after ingestion, include the activity of digestive enzymes and gut microbiota. These processes are complex and it is challenging to ascertain the active structures and interacting targets in the body. The genetic polymorphism of digestion enzymes and the individuality of gut microbiota play an important role transferring in vitro data into in vivo data [159,160]. Gut enzymes, which influence the absorption of polyphenols, are LPH and cytosolic β-glucosidase (CBG) [100,161]. Flavonoids mostly appear as glucosides before being hydrolyzed enzymatically or non-enzymatically in the gut [100]. LPH is located on the luminal side of the enterocytes and hydrolyzes flavonoid-*O*-β-d-glucosides whose aglycones can easily pass the membrane by passive diffusion. The CBG is located within the enterocytes. The polar flavonoid glucoside has to be transported into the cell before it can be hydrolyzed by CBG [100,160,161]. At least two more β-glucosidases, termed glucocerebrosidase and pyridoxine glucoside hydrolase, exist intracellularly [162]. In experiments, the transport of quercetin-glucosides into the enterocytes and blood stream appeared to be faster than of quercetin-aglycones. It is suggested that the glucoside is enabled due to the glucose moiety to act as a substrate of SGLT1 [163,164]. Tsuchihashi et al. investigated the impact of different strains of human microbiota on the intestinal metabolism of flavonoids. Most of the strains performed the hydrolysis of the glucosides to the aglycones except for the aerobe bacteria strains. Some of the bacteria strains appear to catalyze hydrolysis of specific flavonoids rather than others [165]. The main absorption process is located in the small intestine and there is not as much gut bacteria as at lower sections of the gut. Although the impact of microbiota to cleavage of food components is not as big at this point, Schantz et al. showed that the catechin tannins were cleaved by gut microbiota in human ileal fluids. (−)-Epigallocatechin-3-*O*-gallate (EGCG) was metabolized to (−)-epicatechin-3-*O*-gallate (EGC) and gallic acid (GA), EGC to 3’,4’,5’-trihydroxyphenyl-γ-valerolactone and ECG to epicatechin and GA after incubation [166]. Esters of phenyl acrylic acids such as chlorogenic acid are hydrolyzed due to esterase activity of colon microbiota at lower sections of the gut [167,168].

## 8. Conclusions

The inhibition of intestinal LCFA, glucose and fructose transporters is a promising target regarding the prevention and treatment of not only metabolic disorders such as T2DM, but also other diseases which are correlated with high blood concentration of these food components. Traditionally, used plants are very suitable as raw starting material for detecting and identifying new active substances. The plant extract studies, which are listed in Table 4, Table 5 and Table 6, show that not only polyphenols, but also polysaccharids are able to act as intestinal uptake inhibitors of LCFA, glucose and fructose. The analysis of binding sites of relevant intestinal transporters and their interactions with prominent inhibitors is a crucial step in understanding the transport mechanisms and in identifying selective inhibitors. In contrast to intestinal glucose transporters, the binding sites of LCFA and fructose transporters are not as well characterized and examined. Additionally, research has to be done to test more plant extracts for their inhibitory activity on intestinal LCFA and fructose transporters.

Generally, the inhibition of LCFA, glucose and fructose uptake in the intestine causes higher luminal concentrations of these nutrients at lower sections of the gut, which can have different impacts. On the one hand, it can activate the ileal break, which reports to the brain the signal of enough food intake and decreases appetite [169]. On the other hand, unusual osmotic conditions due to unabsorbed monosaccharides and fatty acids and cleavage of these food compounds by enterobacteria can lead to side effects such as flatulence and gripes [170,171]. It is an approach to find uptake inhibitors which leads to a delay of saturated LCFA, glucose and fructose absorption instead of total inhibition. In this case, the ileal break is activated, food intake is decreased and at the same time side effects are reduced compared to total uptake inhibition.

## Figures and Tables

**Table 1:** Predominant tissue expression of the intestinal LCFA, glucose and fructose transporters.

FATP2	FATP4	SGLT1	GLUT2	GLUT5
Tissue expression				
Liver, kidney, intestine [13,14], pancreas, placenta [13]	Small intestine, adipose tissue, brain, liver, skin, heart [14]	Small intestine, kidney, heart, prostate [18]	β-cells, liver, intestine, kidney [19]	Intestine, testis, kidney, skeletal muscle, fat tissue, brain [20]

**Table 2:** Synthetic and plant-based inhibitors: Specific inhibitors of each of the discussed transporters, which could be suitable for cell line experiments to determine transport activity in the presence of other transporters.

FATP2	FATP4	SGLT1		GLUT2	GLUT5
Specific inhibitors					
Grassofermata (I), Lipofermata (II) [13]	4-Aryl-3,4-dihydro- Pyrimidin-2(1H)- ones (III) [54]	Phlorizin (IV) [55,56], LX2761 (V) [57,58]	Phloretin (VI) [59,60]		Astragalin-6-glucoside (VII), N-[4-(methylsulfonyl)- 2-nitrophenyl]- 1,3-benzodioxol-5-amine (MSNBA) (VIII) [53,61]
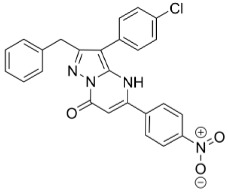	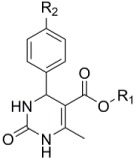 III	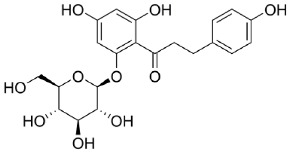	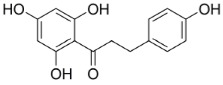 VI		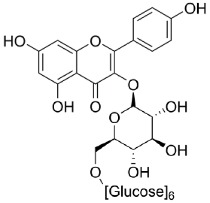
I		IV			VII
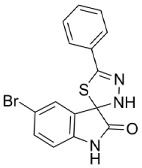		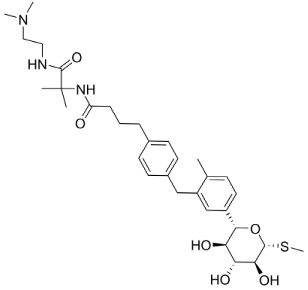			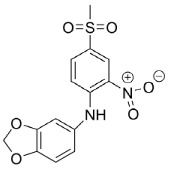
II		V			VIII

**Table 3:** Characteristics of SGLT1 (solute carrier 5 (SLC5) gene family) and GLUT2 (SLC2 gene family). SGLT1 is the predominant glucose transporter in the intestine and it shows complex functionality. Whereas SGLT1 appears to be an active cotransporter, which at first requires the binding of two sodium ions, GLUT2 facilitates the passive diffusion of glucose through the cell membrane. SGLT1 owns a small range of substrate variety and transports only glucose and galactose, whereas GLUT2 transports glucose, galactose, fructose, mannose and glucosamine.

Characteristics of Intestinal Glucose Transporters	SGLT1	GLUT2
Type of transporter	Sodium-dependent active cotransporter	Sodium-independent passive transporter
Family	SLC5 (SLC5A1)	SLC2 (SLC2A2)
Substrates	Glucose, galactose	Glucose, galactose, fructose, mannose, glucosamine
Localization	Apical membrane	Basolateral/apical membrane
Total length of amino acid sequence	664 amino acids	524 amino acids
Number of transmembrane segments	14	12
Affinity to glucose	High (*K_M_* = 0.5–2 mM)	Low (*K_M_* = 17 mM)
Saturation	Yes, >10 mM glucose (capacity low)	No (capacity high)

**Table 4:** In many of the listed studies, plant extracts were tested in different models simultaneously. This table presents plants which were tested for their inhibiting and downregulating effects on intestinal glucose transporters in Caco-2 cells. Whenever inhibition or downregulation of glucose transporters by plant extracts could be observed but the effect was not described precisely, ‘uptake inhibition’ or ‘downregulation’ respectively is placed in the columns. Some plant extracts showed no effects on intestinal transporters in Caco-2 cells under available conditions marked with ‘None’, but changing the preparation method can alter the results as shown for *Camellia sinensis* (L.) Kuntze. ‘Not specified’ means that the information was not mentioned in the study. The discussed active compounds are primarily referred to the transport inhibition, but also to antidiabetic effects in total.

Scientific Plant Name	Part of Plant	Influence on Intestinal Glucose Transporters	Effect on the Expression of Intestinal Glucose Transporters	Discussed Active Compounds	Ref
*Abies balsamea* (L.) Mill.	Bark	None	None	N.s.	[116,117]
*Acanthopanax senticosus* (Rupr. & Maxim.) Harms	Stem bark	Uptake inhibition	N.s.	Isofraxidin, eleutherosides, senticosides, chlorogenic acid	[118]
*Adenophora tryphilla var. japonica* (Regel.) Hara	N.s.	Uptake inhibition	N.s.	N.s.	[119]
*Alnus incana (L.) * Moench	Bark	Uptake inhibition	None	Oregonin	[116,117]
*Angelica gigas* Nakai	N.s.	Uptake inhibition	N.s.	N.s.	[119]
*Astragalus membranaceus* (Fisch.) Bunge	N.s.	Uptake inhibition	N.s.	N.s.	[119]
*Avena*L. sp.	Grains	Uptake inhibition	N.s.	β-glucans, phenolic acids (caffeic, gallic, *p*-coumaric, ferulic and sinapic acid), flavonoids, lignans, avenanthramides (AVE A, AVE B, AVE C)	[120]
*Camellia sinensis* (L.) Kuntze	N.s. ^1^	Uptake inhibition	N.s.	Catechins (epicatchin gallate)	[121]
*Camellia sinensis* (L.) Kuntze	Leaf	Uptake inhibition	N.s.	(−)-epigallocatechin gallate, (−)-epigallocatechin, (−)-epicatechin, (+)-catechin	[122]
*Camellia sinensis* (L.) Kuntze	Leaf	None	N.s.	Catechins, theaflavins, caffeine, polysaccharides	[123]
*Capsella bursa-pastoris* (L.) Medik.	N.s.	Uptake inhibition	N.s	N.s.	[119]
*Capsicum annuum* L.	N.s.	Uptake inhibition	N.s.	N.s.	[119]
*Cinnamomum camphora* (L.) J.Presl	N.s.	Uptake inhibition	N.s.	N.s.	[119]
*Citrus junos* Siebold ex Tanaka	N.s.	Uptake inhibition	N.s.	N.s.	[119]
*Citrus paradisi* Macfad	N.s.	Inhibition of SGLT1	N.s.	kaempferol rutinoside, naringenin-7-*O*-rutinoside	[124]
*Citrus unshiu* (Yu.Tanaka ex Swingle) Marcow	N.s.	Uptake inhibition	N.s.	N.s.	[119]
*Codonopsis lanceolata* (Siebold & Zucc.) Benth. & Hook.f. ex Trautv.	N.s.	Uptake inhibition	N.s.	N.s.	[119]
*Cornus officinalis* Siebold & Zucc.	N.s.	Uptake inhibition	N.s.	N.s.	[119]
*Crataegus pinnatifida var. typica* C.K.Schneid.	N.s.	Uptake inhibition	N.s.	N.s.	[119]
*Cuscuta japonica* Choisy	N.s.	Uptake inhibition	N.s.	N.s.	[119]
*Daucus carota ssp. sativus var. atrorubens* Alef.	Root	Uptake inhibition	N.s.	Anthocyanins (cyanidin-3-xylosyl-(feruloylglucosyl)-Galactoside, chlorogenic acid	[125]
*Dendranthema morifolium* (Ramat.) Tzvelev	N.s.	Moderate inhibition of SGLT1 and GLUT2	N.s.	1,3-dicaffeoylquinic acid, 5-caffeoylquinic acid, 1,5-dicaffeoylquinic acid, 3,5-dicaffeoylquinic acid	[126]
*Diospyros kaki* L.f.	N.s.	Uptake inhibition	N.s.	N.s.	[119]
*Eucommia ulmoides* Oliv.	Leaf	Uptake inhibition	N.s.	Lignans, iridoids, polyphenols (catechin, caffeic acid, 2,6-dihydroxy-benzoic acid, mandelic acid), steroids, triterpenes, organicacids, polysaccharides, flavonoids, amino acids	[127]
*Eucommia ulmoides* OLIV.	N.s.	Uptake inhibition	N.s.	N.s.	[119]
*Fragaria*L. sp. *‘Albion’*	Fruit	Predominant inhibition of GLUT2	N.s.	Polyphenols (pelargonidin-3-*O*-glucoside), phenolic acids and tannins	[128]
*Gaultheria hispidula* (L.) Muhl. Ex Bigelow	N.s.	Uptake inhibition	None	N.s.	[116]
*Ginkgo biloba* L.	N.s.	Uptake inhibition	N.s.	N.s.	[119]
*Helichrysum italicum* (ROTH) G. Don	N.s.	Inhibition of SGLT1	N.s.	kaempferol-3-*O*-glucoside, chlorogenic acid-3-*O*-glucoside, naringenin-7-*O*-glucoside, naringenin diglycoside	[124]
*Ipomoea batatas* (L.) Lam.	Stem	Moderate inhibition of SGLT1 and GLUT2	N.s.	5-caffeoylquinic acid, 3,5-dicaffeoylquinic acid	[126]
*Juniperus communis* L.	N.s.	Uptake inhibition	None	N.s.	[116]
*Kalmia angustifolia* L.	N.s.	None	None	N.s.	[116]
*Larix laricina* (Du Roi) K.Koch	Bark	Mild to moderate inhibition	None	N.s.	[116,117]
*Lonicera japonica* Thunb.	N.s.	None	N.s.	5-caffeoylquinic acid,	[126]
*Lycium barbarum* L.	N.s.	Uptake inhibition	Downregulation of SGLT1	Polysaccharides (average weight of 10 to 30 kDa)	[129]
*Lycopodium clavatum* L.	N.s.	Uptake inhibition	None	N.s.	[116]
*Malpighia emarginata* DC.	Fruit	Uptake inhibition	N.s.	cyanidin-3-α-*O*- rhamnoside, pelargonidin-3-α-*O*-rhamnoside, Quercetin-3-α-*O*-rhamnoside	[130]
*Malus domestica* Borkh. *‘Golden delicious’*	Fruit	Predominant inhibition of GLUT2	N.s.	Polyphenols (Quercetin-3-*O*-rhamnoside, phlorizin) phenolic acids (5-caffeoylquinic acid) and tannins	[128]
*Matricaria recutita* L.	N.s.	Predominant inhibition of GLUT2	N.s.	Apigenin-7-*O*-glucoside, apigenin	[122]
*Mentha arvensis* L. *var. japonica*	N.s.	Uptake inhibition	N.s.	N.s.	[119]
*Musa x sapientum* L.	Infructescence stalks	Indirect Inhibition caused by reduction of the Na^+^-gradient due to the decrease of Na^+^/K^+^-ATPase activity	None	cycloartenol, cycloeucalenol, 24-methylene cycloartanol, campesterol, β-sitosterol and stigmasterol, serotonin and norepinephrine	[131]
*Opuntia ficus-indica* (L.) Mill.	N.s.	Uptake inhibition	N.s.	N.s.	[119]
*Panax ginseng* C.A.Mey	Root	Inhibition of SGLT1	N.s.	Protopanaxadiol-type Ginsenosides (Rd, Rg_3_, Rh_2_, F_2_, compound K)	[132]
*Panax notoginseng* (Burkill) F.H.Chen	Root	Uptake inhibition	Downregulation of SGLT1	Protopanaxatriol ginsenoside Rg1	[133]
*Picea glauca* (Moench) Voss	Needle, cone, bark.	Uptake inhibition	None	Phenolic acids, stilbene, flavonoids	[116,117]
*Picea mariana* (Mill.) Britton, Sterns & Poggenb.	N.s.	Uptake inhibition	Downregulation of GLUT2	N.s.	[116]
*Pinus banksiana*Lamb.	N.s.	Uptake inhibition	None	N.s.	[116]
*Pinus pinea* L.	Bark	Predominant inhibition of SGLT1	Downregulation of SGLT1 and GLUT2	N.s.	[134]
*Polygonatum odoratum* (Mill.) Druce	Root	Uptake inhibition	N.s.	Sappanin-type homoisoflavonoids (5,7-dihydroxy-3-(4′-hydroxybenzyl)-6-methylchroman-4-one (EA-1), 5,7-dihydroxy-3-(4′-hydroxybenzyl)-6-methyl-8-methoxychroman-4-one (EA-2), and 5,7-dihydroxy-3-(4′-hydroxybenzyl)-6, 8-dimethylchroman-4-one (EA-3)	[135]
*Populus balsamifera* L.	N.s.	Uptake inhibition	None	N.s.	[116,117]
*Pueraria thunbergiana* (Siebold & Zucc.) Benth	N.s.	Uptake inhibition	N.s.	N.s.	[119]
*Punica granatum* L.	Fruit	Inhibition of SGLT1	Downregulation of SGLT1	Polyphenols (anthocyanins, hydrolysable tannins)	[119]
*Punica granatum* L. *‘Mollar’*	Fruit	None (in Caco-2 model)	None (in Caco-2 model)	Punicalagin, Punicalin, ellagic acid	[136]
*Prunella vulgaris* L.	N.s.	None	Downregulation	N.s.	[137]
*Prunus armeniaca* L. *var. ansu*	N.s.	Uptake inhibition	N.s.	N.s.	[119]
*Psidium guajava* L.	Leaf, fruit	Inhibition of SGLT1 and GLUT2	N.s.	Phloridzin, phloretin, quercetin, quercitrin, isoquercitrin, hyperoside, avicularin, guaijaverin, procyanidin B1, B2, (+)-catechin, (−)-epicatechin, gallocatechin, epicatechin gallate, gallic and ellagic acid	[138]
*Rhododendron groenlandicum* (Oeder) Kron & Judd	Leaf	Moderate to strong inhibition	None	N.s.	[116,117]
*Rhododendron tomentosum* Harmaja	N.s.	Strong inhibition	Downregulation of SGLT1	N.s.	[116,117]
*Raphanus sativus* L.	N.s.	Uptake inhibition	N.s.	N.s.	[119]
*Rosa canina* L.	Seed	Inhibition of SGLT1 and GLUT2	N.s.	Tiliroside	[139]
*Rosa laevigata* Michx.	N.s.	Uptake inhibition	N.s.	N.s.	[119]
*Rosa rugosa* Thunb.	N.s.	Uptake inhibition	N.s.	N.s.	[119]
*Rubus coreanus* Miq.	N.s.	Uptake inhibition	N.s.	N.s.	[119]
*Salix planifolia* Pursh	N.s.	Uptake inhibition	None	N.s.	[116]
*Saposhnikovia divaricata* (Turcz.) Schischk.	N.s.	Uptake inhibition	N.s.	N.s.	[119]
*Sarracenia purpurea* L.	N.s.	Uptake inhibition	None	N.s.	[116,117]
*Solanum lycopersicum* L.	Seed	Uptake inhibition	Downregulation of GLUT2	Saponins (Tomatoside A)	[140]
*Sorbus decora* (Sarg.) C.K.Schneid.	N.s.	Moderate inhibition	None	N.s.	[116,117]
*Terminalia chebula* Retz.	Fruit	None	N.s.	Chebulagic acid	[141]
*Triticum* L. sp.	Wheat aleurone	Uptake inhibition	N.s.	Ferulic acid, feruloylated arabinoxylan mono- and oligosaccharides	[142]
*Vaccinium vitis-idaea* L.	Fruit	Uptake inhibition	None	N.s.	[116]
*Vitis labrusca*L. ‘Concord’	Fruit	Uptake inhibition	Downregulation of GLUT2	Flavan-3-ols, flavonols (quercetin), stilbenes (resveratrol), phenolic acids (gallic, caffeic acids), anthocyanins (cyanidin-3,5-*O*-diglucoside delphinidin-3-*O*-glucoside)	[143]
*Vitis labrusca*L. ‘Niagara’	Fruit	Uptake inhibition	Downregulation of GLUT2	Flavan-3-ols, flavonols (quecetin), stilbenes (resveratrol), phenolic acids (gallic, caffeic acids)	[143]
*Vitis vinifera* L.	Grape skin	Uptake inhibition	Upregulation of GLUT2	Malvidin-3-glucose	[144]
*Zea mays* L.	Corn bran	Uptake inhibition	N.s.	Ferulic acid, feruloylated arabinoxylan mono- and oligosaccharides	[142]
*Ziziphus jujuba var. inermis* (Bunge) Rehder	N.s.	Uptake inhibition	N.s.	N.s.	[119]
Mixture of *Gymnema sylvestre* (Retz.) R. Br. Ex Sm., *Coffea* L. sp., *Vitis* L. sp., *Hibiscus* L. sp., *Cinnamomum* Schaeff. sp., *Ocimum* L. sp., *Artemisia dracunculus* L., *Zingiber* Mill. sp., *Curcuma* L. sp.	N.s.	Predominant inhibition of GLUT2	N.s.	flavonoid aglycones (quercetin, kaempferol), caffeic acid, and *p*-coumaric acid	[145]
*Mixture of Ilex latifolia*Thunb. and *Camellia sinensis* (L.) Kuntze *var. sinensis*	N.s.	Strong inhibition of SGLT1 and GLUT2	N.s.	Chlorogenic acids (5-caffeoylquinic acid, 3,5-dicaffeoylquinic acid), (–)-epicatechin gallate, (–)-epigallocatechin gallate, flavonols, flavonol glycosides (rutin)	[126]
Mixture of *Vaccinium* L. sp., Sambucus L. sp., *Rubus* L. sp., *Fragaria* L. sp.	Fruit, Seed	Inhibition of SGLT1 and GLUT2	Downregulation of SGLT1 and GLUT2	Flavonoids, anthocyanins (cyanidin-3-glucoside, cyanidin-3-rutinoside)	[146]

**^1^** Not specified.

**Table 5:** In some of the listed studies, plant extracts were tested in different models simultaneously. This table presents plants which were tested for their inhibiting and downregulating effects on intestinal fructose transporters in Caco-2 cells. Whenever inhibition or downregulation of fructose transporters by plant extracts could be observed but the effect was not described precisely, ‘uptake inhibition’ or ‘downregulation’ respectively is placed in the columns. ‘Not specified’ means that the information was not mentioned in the study. The discussed active compounds are primarily referred to the transport inhibition, but also to antidiabetic effects in total.

Scientific Plant Name	Part of Plant	Influence on Intestinal Fructose Transporters	Effect on the Expression of Intestinal Fructose Transporters	Discussed Active Compounds	Ref
*Allium cepa* L.	N.s. ^1^	N.s.	N.s.	N.s.	[147]
*Camellia sinensis* (L.) Kuntze	Leaf	Uptake inhibition	N.s.	(−)-epigallocatechin gallate, (−)-epigallocatechin, (−)-epicatechin, (+)- catechin	[122]
*Chrysanthemum* L. *sp.*	N.s.	N.s.	N.s.	N.s.	[147]
*Curcuma longa* L.	N.s.	Uptake inhibition	N.s.	Curcumin, bisdemethoxycurcumin, dimethoxycurcumin	[147]
*Glycine max* (L.) Merr.	N.s.	N.s.	N.s.	N.s.	[147]
*Matricaria recutita* L.	N.s.	Predominant inhibition of GLUT2	N.s.	Phenolic glucosides (apigenin-7-*O*-glucoside, apigenin)	[122]
*Myrica* L. *sp.*	Bark	N.s.	N.s.	N.s.	[147]
*Panax ginseng* C.A. Mey.	N.s.	N.s.	N.s.	N.s.	[147]
*Passiflora* L. *sp.*	N.s.	N.s.	N.s.	N.s	[147]
*Psidium guajava* L.	Leaf	Uptake inhibition	N.s.	Quercetin, catechin	[147]
*Rosmarinus officinalis* L.	N.s.	Uptake inhibition	N.s.	N.s.	[147]
*Vitis labrusca* L. *‘Concord’*	Fruit	Uptake inhibition	Downregulation of GLUT2	Flavan-3-ols, flavonols (quercetin), stilbenes (resveratrol), phenolic acids (gallic, caffeic acids), anthocyanins (cyanidin-3,5-*O*-diglucoside delphinidin-3-*O*-glucoside)	[143]
*Vitis labrusca* L. *‘Niagara’*	Fruit	Uptake inhibition	Downregulation of GLUT2	Flavan-3-ols, flavonols (quercetin), stilbenes (resveratrol), phenolic acids (gallic, caffeic acids)	[143]

^1^ Not specified.

**Table 6:** Two plant extracts were tested and both showed inhibitory and downregulating activity on intestinal fatty acid transporters and their proteins when tested in Caco-2 cells. The discussed active compounds are primarily referred to the transport inhibition, but also to antidiabetic effects in total.

Scientific Plant Name	Part of Plant	Influence on Intestinal Fatty Acid Transporters	Effect on the Expression of Intestinal Fatty Acid Transporters	Discussed Active Compounds	Ref
*Avena* L. *sp.*	Grains	Uptake inhibition	Downregulation of FABP and FATP4	*Β*-glucan	[148]
*Hordeum* L. *sp.*	Grains	Uptake inhibition	Downregulation of FABP and FATP4	*Β*-glucan	[148]
