# Correction: Schreck, K. and Melzig, M.F. Intestinal Saturated Long-Chain Fatty Acid, Glucose and Fructose Transporters and their Inhibition by Natural Plant Extracts in Caco-2 Cells. *Molecules*, 2018, *23*, 2544

**DOI:** 10.3390/molecules23123290

**Published:** 2018-12-11

**Authors:** Katharina Schreck, Matthias F. Melzig

**Affiliations:** Institute of Pharmacy-Pharmaceutical Biology, Freie Universitaet Berlin, Koenigin-Luise-Str. 2+4, D-14195 Berlin, Germany; kschreck@zedat.fu-berlin.de

The authors wish to make the following correction to their paper [1]:

We have found the following error in Figure 4a and Figure 4b of this article, which was recently published in *Molecules* as part of the Special Issue entitled “Natural Products in Prevention and Treatment of Metabolic Syndrome”. The correct order should be: Figure 4a is supposed to be Figure 4b and Figure 4b is supposed to be Figure 4a. The captions will match after changing the images. Thus, the originally published figures, shown below: 
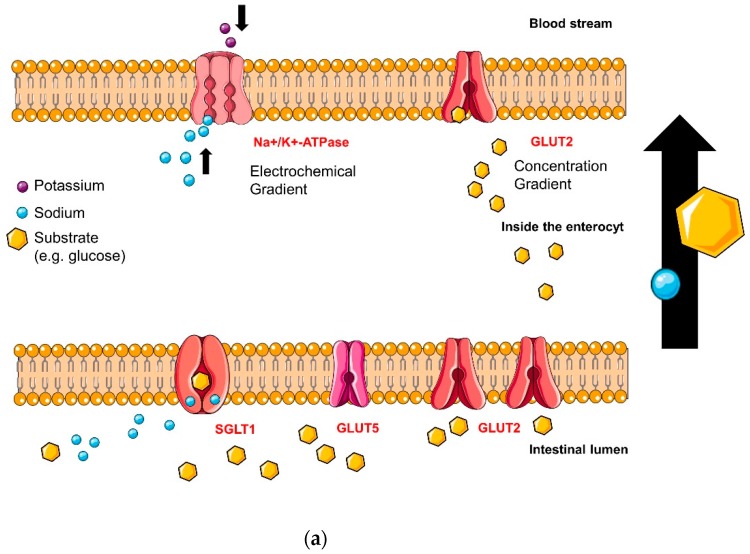
 
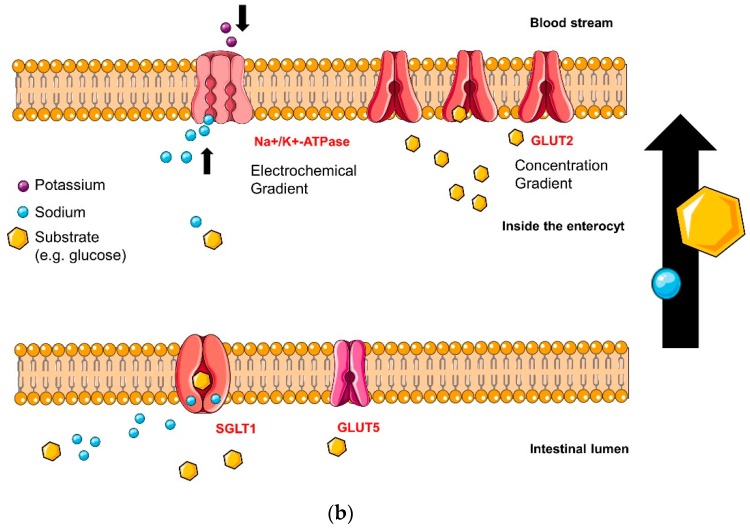
 would be replaced with the following figures, now in the correct order: 
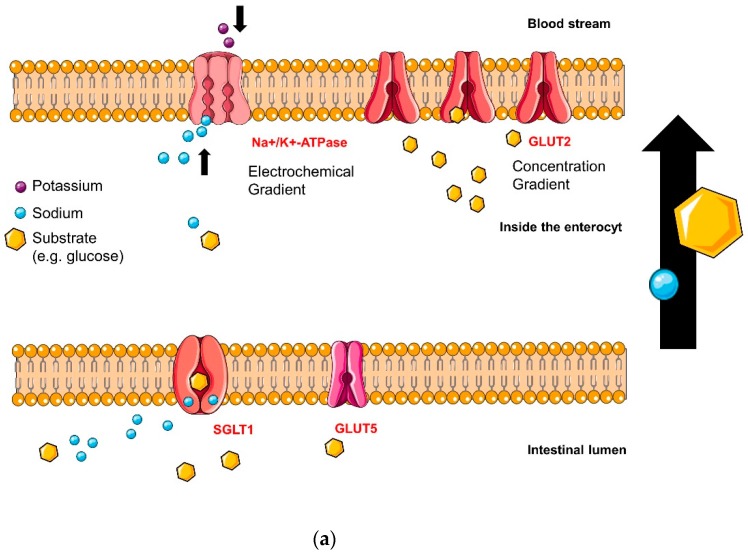
 
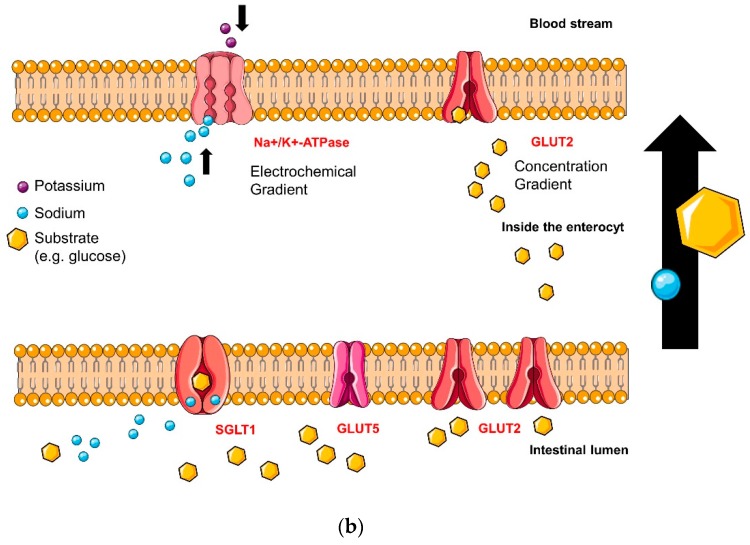


The authors would like to apologize for any inconvenience caused to the readers by these changes.
